# The nociceptin/orphanin FQ receptor system as a target to alleviate cancer‐induced bone pain in rats: Model validation and pharmacological evaluation

**DOI:** 10.1111/bph.14899

**Published:** 2020-01-21

**Authors:** Sonny H.J. Sliepen, Johanna Korioth, Thomas Christoph, Thomas M. Tzschentke, Marta Diaz‐delCastillo, Anne‐Marie Heegaard, Kris Rutten

**Affiliations:** ^1^ Grünenthal Innovation Grünenthal GmbH Aachen Germany; ^2^ Department of Drug Design and Pharmacology, Faculty of Health and Medical Sciences University of Copenhagen Copenhagen Denmark

## Abstract

**Background and Purpose:**

Cancer‐induced bone pain remains inadequately controlled, and current standard of care analgesics is accompanied by several side effects. Nociceptin/orphanin FQ peptide (NOP) receptor agonists have demonstrated broad analgesic properties in rodent neuropathic and inflammatory pain models. Here, we investigate the analgesic potential of NOP receptor activation in a rodent cancer‐induced bone pain model.

**Experimental Approach:**

Model validation by intratibial inoculation in male Sprague Dawley rats was performed with varying MRMT‐1/Luc2 cell quantities (0.5–1.5 × 10^6^·ml^−1^) and a behavioural battery (>14 days post‐surgery) including evoked and non‐evoked readouts: paw pressure test, cold plate, von Frey, open field, and weight distribution. Anti‐allodynic potential of the endogenous NOP receptor ligand nociceptin (i.t.) and NOP receptor agonist Ro65‐6570 ( i.p.) was tested using von Frey filaments, followed by a combination experiment with Ro65‐6570 and the NOP receptor antagonist J‐113397 (i.p.). Plasma cytokine levels and NOP receptor gene expression in dorsal root ganglion (DRG, L4‐L6) and bone marrow were examined.

**Key Results:**

Inoculation with 1.5 × 10^6^·ml^−1^ of MRMT‐1/Luc2 cells resulted in a robust and progressive pain‐related phenotype. Nociceptin and Ro65‐6570 treatment inhibited cancer‐induced mechanical allodynia. J‐113397 selectively antagonized the effect of Ro65‐6570. MRMT‐1/Luc2‐bearing animals demonstrated elevated plasma cytokine levels of IL‐4, IL‐5, IL‐6 and IL‐10 plus unaltered NOP‐r gene expression in DRG and reduced expression in bone marrow.

**Conclusion and Implications:**

Nociceptin and Ro65‐6570 selectively and dose‐dependently reversed cancer‐induced bone pain‐like behaviour. The NOP receptor system may be a potential target for cancer‐induced bone pain treatment.

**LINKED ARTICLES:**

This article is part of a themed issue on The molecular pharmacology of bone and cancer‐elated bone diseases. To view the other articles in this section visit http://onlinelibrary.wiley.com/doi/10.1111/bph.v178.9/issuetoc

**Abbreviations**
DRGs: dorsal root ganglions
Luc2: luciferase
NOP receptor: nociceptin/orphanin FQ opioid peptide receptor
oprl1: opioid‐related nociceptin receptor 1
Tbp: TATA‐binding box protein
TRPV1: transient receptor potential cation channel subfamily V member 1

**What is already known**

- Current treatment for bone cancer pain is inadequate and represents an unmet medical need.
- Nociceptin/orphanin FQ (NOP) receptor agonists effectively alleviate inflammatory and neuropathic pain in multiple preclinical models.

**What does this study adds**

- NOP receptor agonists selectively and effectively attenuate mechanical allodynia in a bone cancer pain model.

**What is the clinical significance**

- The NOP receptor system represents a promising novel target for treatment of bone cancer pain

## 1 INTRODUCTION

The most common type of pain in cancer patients is bone cancer pain (O'Toole & Boland, 2006), which has a nociceptive and neuropathic component (Colvin & Fallon, 2008; O'Toole & Boland, 2006). In 1986, the World Health Organization established a three‐step guideline for adequate malignant pain treatment, starting with non‐opioids (Step 1) for mild pain, weak opioids ± non‐opioids and adjuvants for mild to moderate pain (Step 2), and strong opioids ± non‐opioids and adjuvants for moderate to severe pain (Step 3; WHO, 2018; Zhu et al., 2015). In particular, for bone pain, it is recommended to combine treatment for pain with radiotherapy and bisphosphonates (WHO, 2018). However, in 43.4% of patients, the cancer was undertreated according to the pain management index (Greco et al., 2014) and 20% of patients rotate through ≥3 opioids before an efficient balance between analgesia and side effects was reached (Sloan, 2008). Nonetheless, implementation of the guidelines has been shown to reduce the most frequently occurring symptoms, for example, impaired activity, mood changes, constipation, nausea and dry mouth (Meuser et al., 2001). Nevertheless, opioid use unwanted effects are still a risk accompanying long‐term treatment of chronic pain patients with opioids (Kaye et al., 2017). This has contributed to the current opioid crisis in the United States and constitutes a major health concern as increasing numbers of opioid‐related deaths are reported (Koenig, 2018). The opioid crisis in combination with the unmet medical need for a more efficacious and better tolerable treatment specifically for malignant pain is an important current driver for the development of novel analgesic compounds.

The nociceptin/orphanin FQ opioid peptide (NOP) receptor is the fourth (besides μ, δ, and κ) opioid receptor (Meunier et al., 1995; Reinscheid et al., 1995), spinal or peripheral activation exerts anti‐nociceptive effects, while supraspinally it acts in a pro‐nociceptive fashion in rodents (Schroder, Lambert, Ko, & Koch, 2014). Spinal and systemic administration of NOP receptor agonists are highly efficacious and potent in attenuating symptoms of neuropathic and inflammatory pain (Courteix et al., 2004; Fu, Wang, & Wu, 2006; Ma, Xie, Dong, Wang, & Wu, 2003; Schiene, Tzschentke, Schroder, & Christoph, 2015). Moreover, activation of the NOP receptor has anti‐rewarding and anti‐abuse effects in rodents (Ciccocioppo, Angeletti, Sanna, Weiss, & Massi, 2000; Lin & Ko, 2013; Marquez, Nguyen, Hamid, & Lutfy, 2008; Rutten, De Vry, Bruckmann, & Tzschentke, 2010; Rutten, De Vry, Bruckmann, & Tzschentke, 2011) making the NOP receptor system an interesting target for pain management with potentially lower abuse potential.

The aim of the present study was to investigate the putative analgesic effect of NOP receptor activation in the MRMT‐1 rat model of cancer‐induced bone pain. Intratibial inoculation with rat mammary gland carcinoma (MRMT‐1) cells was previously described as a robust translational model for metastatic cancer‐induced bone pain in rats (Medhurst et al., 2002). To validate the robustness of our model, the pain‐like phenotype induced by different MRMT‐1 cell concentrations was examined. Additionally, the inflammatory component of the model was assessed by plasma cytokine levels and NOP receptor gene expression in dorsal root ganglions (DRGs) and, for the first time, in bone marrow. Thereafter a battery of behavioural tests was conducted in order to determine which readout is best suited to assess pain‐related behaviour in this model. Next, the role of the NOP receptor in cancer‐induced bone pain was investigated by intrathecal administration of the endogenous ligand (nociceptin) and systemic administration of a small‐molecule NOP receptor agonist (Ro65‐6570) alone, or in combination with a NOP receptor antagonist (J‐113397).

## 2 METHODS

### 2.1 Animals

A total of 284 four‐week‐old male Sprague Dawley rats (100–124 g; Janvier Laboratories, Le Genest St Isle, FR) were housed in groups of four in individually ventilated 1,500 U cages (1,500 cm^2^, Tecniplast, Italy) with Lignocel Flake J. bedding (J. Rettenmaier & Söhne GmbH & Co. KG, Rosenberg, DE) in a temperature‐controlled room (22 ± 2°C), on a 12/12 light/dark cycle (lights on at 06:00 a.m.). Water and food (Ratte/Maus‐Haltung, Ssniff, Soest, DE) were provided ad libitum, and a pure Aspen medium wood block (Ssniff, Soest, DE) was provided as environmental enrichment. Rats were left to acclimatize to the facility for 1 week prior to initiation of experiments and animal welfare, for example, body weight, grooming, posture, and gait, was assessed on a daily basis. All experiments were performed according to the German Animal Welfare Act and were approved by the local government authority (LANUV AZ. No. 81‐02.05.40.17.087). Animal studies are reported in compliance with the ARRIVE guidelines (Kilkenny, Browne, Cuthill, Emerson, & Altman, 2010) and with the recommendations made by the *British Journal of Pharmacology.*

### 2.2 Study design

**FIGURE 1:**
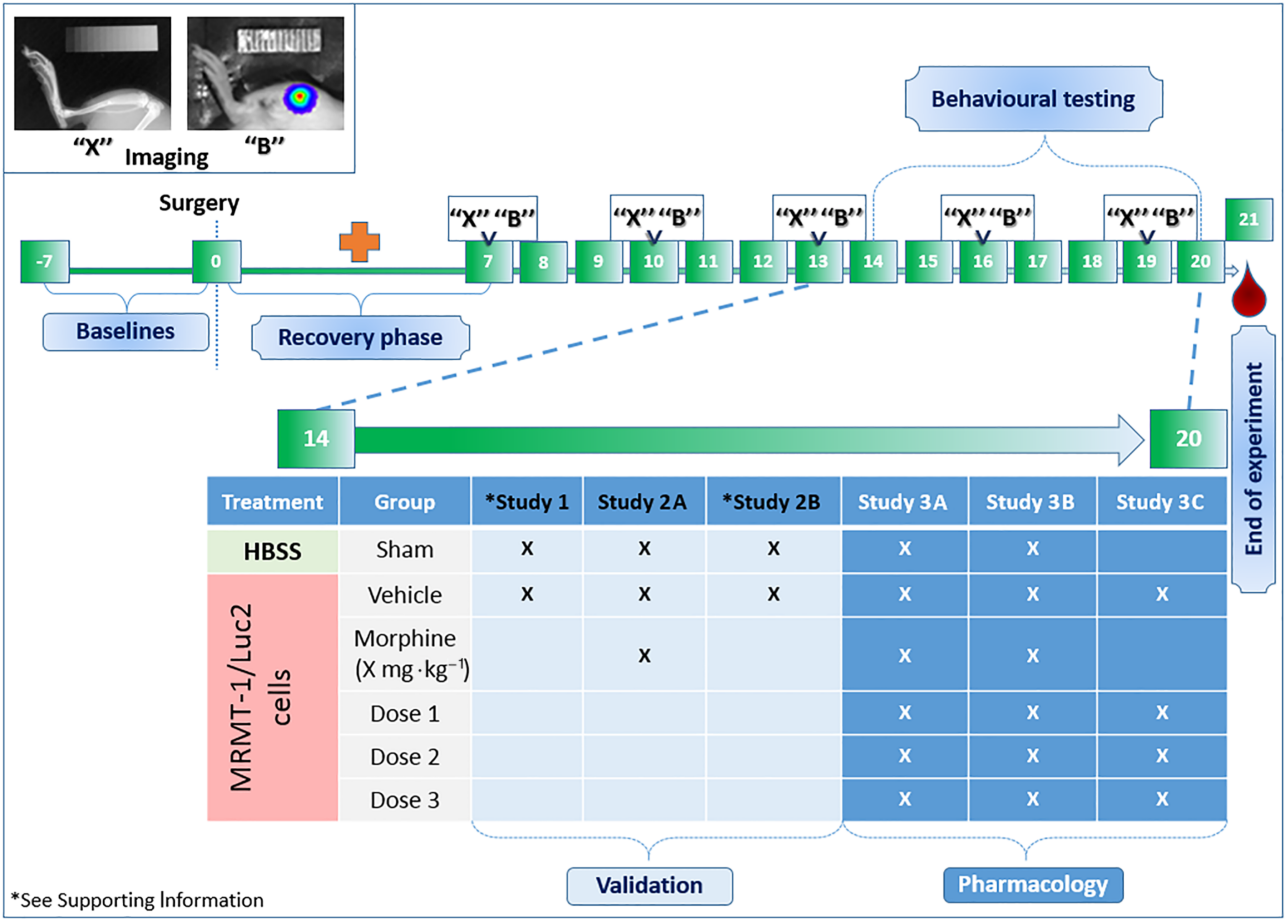
A brief schematic representation of the experimental design. Generally, prior to surgery (Day 0) training and baseline measurements were performed. Thereafter, animals were allowed to recover for 1 week (red cross) in which no measurements occurred, except for the limb use test, which was performed on a daily basis until day 20 post‐surgery (not shown in figure). X‐ray densitometry images (“X”) and monitoring of bioluminescence signal (“B”) to examine bone degradation and tumour growth progression, respectively, were obtained on Days 7, 10, 13, 16, and 19 post‐surgery. Animals were subjected to behavioural testing after the second week on Days 14 to 20 post‐surgery, exact days depending on the tests, for example, evoked or non‐evoked readouts. Blood was obtained or tissue was harvested at the end of the experiment, 21 days post‐surgery. The table shows which animal treatment group was included (X) per study. Sham animals were included in each study for behavioural testing, except Study 3C; vehicle‐treated animals were always included. Results of the model validation studies “Validation”: Study 1 is shown in appendices; Study 2A is shown in Figure 2; Study 2B is shown in Table 2; Results of the pharmacological interventions with NP agonists “Pharmacology”: Study 3A–C is shown in Figure 3

All studies were conducted between January 2017 and September 2018. Behavioural testing occurred between 07:00 am and 01:00 pm and X‐ray and bioluminescence imaging was performed afterwards. In case of multiple measurements per day, the order was limb use first, then experimental test and, finally, imaging. All experiments are reported in accordance with the ARRIVE guidelines (Kilkenny et al., 2010). Group sizes were designed to be equal but did eventually vary due to the applied exclusion criteria (Figure 1).

The first study (Study 1, total *n* = 60) was aimed to determine which quantity of intratibially inoculated MRMT‐1/Luc2 cells resulted in a robust cancer‐induced bone pain‐like phenotype. Therefore, three concentrations of MRMT‐1/Luc2 cells (i.e., 0.5 × 10^6^·ml^−1^, 1.0 × 10^6^·ml^−1^, and 1.25 × 10^6^·ml^−1^) were inoculated and mechanical allodynia (von Frey hair) was tested (16–17 days post‐surgery). Additionally, X‐ray densitometry and bioluminescence signal images were obtained 7, 10, 13, 16 and 19 days post‐surgery and post‐mortem tumour presence was analysed (see Figure S1 and Data S1). The first study also includes characterization of the model by analysis of inflammatory mediators (Table 1) and ipsilateral DRGs L4‐L6 and ipsilateral bone marrow were harvested 21 days post‐surgery for NOP receptor (*oprl1*) gene expression analysis (Table 2).

**TABLE 1:** Levels of inflammatory markers measured in plasma of sham and 1.5 × 10^6^·ml^−1^ of MRMT‐1/Luc2‐bearing animals

Inflammatory markers		
Cytokine marker (pg·mol^−1^)	Sham	MRMT‐1/Luc2
IFN‐γ	1.93 ± 0.26	2.85 ± 0.31
IL‐10	68.92 ± 2.0	81.70 ± 4.61*
IL‐13	18.8 ± 0.45	20.80 ± 1.13
IL‐1β	3.65 ± 3.60	2.75 ± 1.30
IL‐4	9.88 ± 0.17	12.02 ± 0.77*
IL‐5	24.92 ± 3.67	57.65 ± 13.46*
IL‐6	494.6 ± 31.34	622.20 ± 43.89*
KC‐GRO	76.21 ± 12.41	118.30 ± 35.05
TNF‐α	7.74 ± 1.29	10.22 ± 0.63

*Note.* Samples measured by MesoScale. Data are represented as mean ± SEM; *n* for sham = 6 and *n* for MRMT‐1/Luc2‐bearing animals = 5.

* *P* < .05 versus sham.

**TABLE 2:** Gene expression of *oprl1* in dorsal root ganglion (DRG) and bone marrow, measured 21 days post‐surgery in sham and 1.5 × 10^6^·ml^−1^ of MRMT‐1/Luc2‐bearing animals

Oprl1 gene expression						
	DRG			Bone marrow		
	Average CT Mean ± SEM		ΔCT Mean ± SEM	Average CT Mean ± SEM		ΔCT Mean ± SEM
	Tbp	oprl1	Tbp–oprl1	Tbp	oprl1	Tbp–oprl1
Sham	29.04 ± 0.20	27.44 ± 0.27	1.60 ± 0.09	27.50 ± 0.22	34.41 ± 0.64	−6.91 ± 0.35
MRMT‐1/Luc2	28.62 ± 0.18	27.05 ± 0.18	1.57 ± 0.05	27.06 ± 0.14	35.80 ± 0.40	−8.74 ± 0.24

*Note.* In DRG, *n* for sham and *n* for MRMT‐1/Luc2‐bearing animals = 10; in bone marrow, *n* for sham and *n* for MRMT‐1/Luc2‐bearing animals = 5.

**FIGURE 2:**
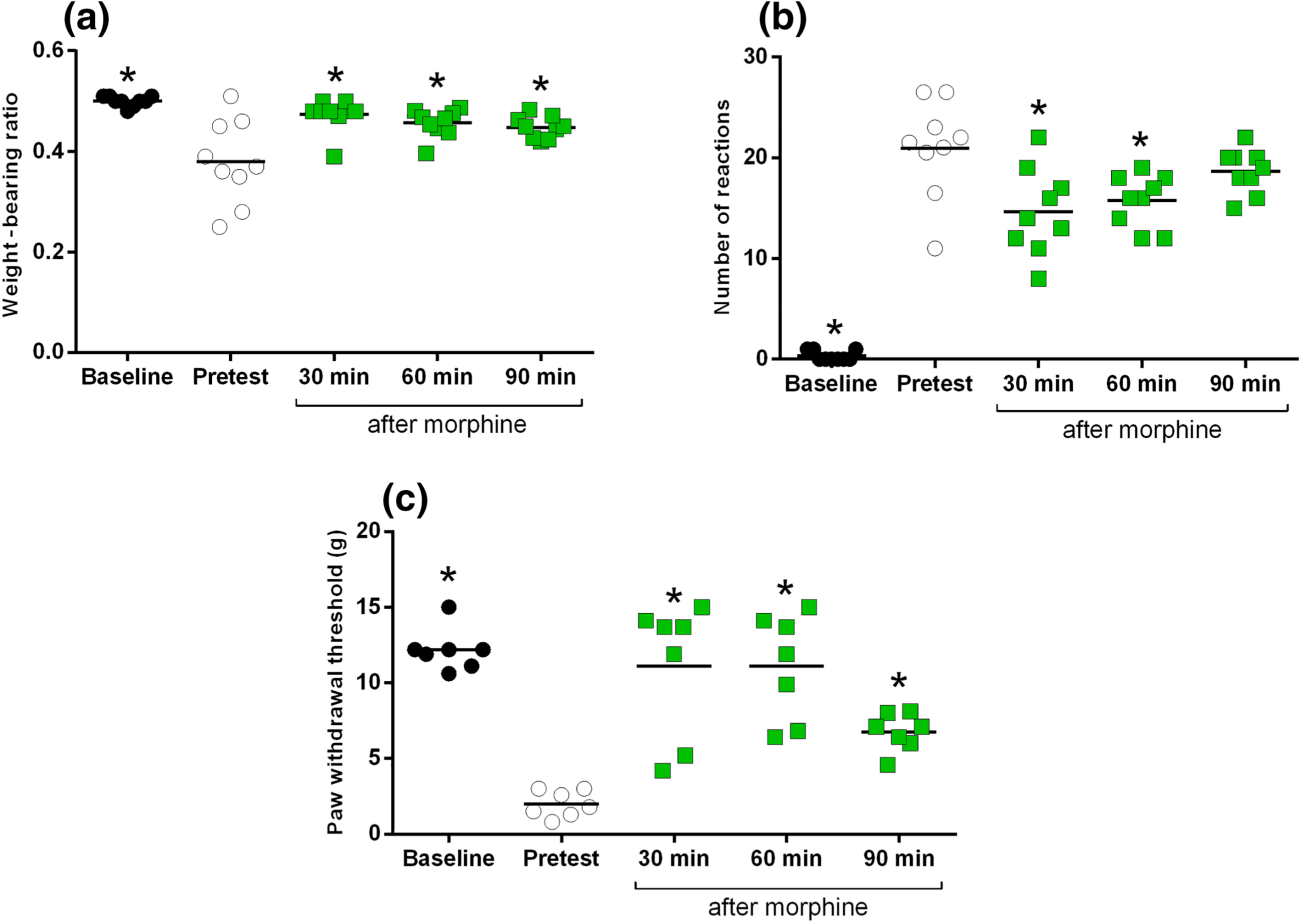
Effect of 1.25 × 10^6^·ml^−1^ of MRMT‐1/Luc2 inoculated cells on (a) weight‐bearing ratio, (b) cold allodynia, and (c) mechanical allodynia, including the effect of i.p. morphine (3.16 mg·kg^−1^) administration. All data are presented as mean ± SEM; *n* = 9, all MRMT‐1/Luc2‐bearing animals; *P < .05 versus pretest

The second experiment (Study 2, total *n* = 58) was designed to determine the feasibility of different behavioural readouts to show a nociceptive phenotype after inoculation of 1.25 × 10^6^·ml^−1^ of MRMT‐1/Luc2 cells and to determine which readouts have a test window allowing the testing of analgesics. In Study 2A von Frey, weight bearing and cold plate test were conducted on Days 15, 17, and 20 post‐surgery, respectively, following i.p. administration of 3.16 mg·kg^−1^ of morphine (Figure 2). In Study 2B, paw pressure and open field test were conducted on Days 16 and 18 post‐surgery respectively (see Table S1 and corresponding methods). All behavioural tests included a baseline measurement.

**FIGURE 3:**
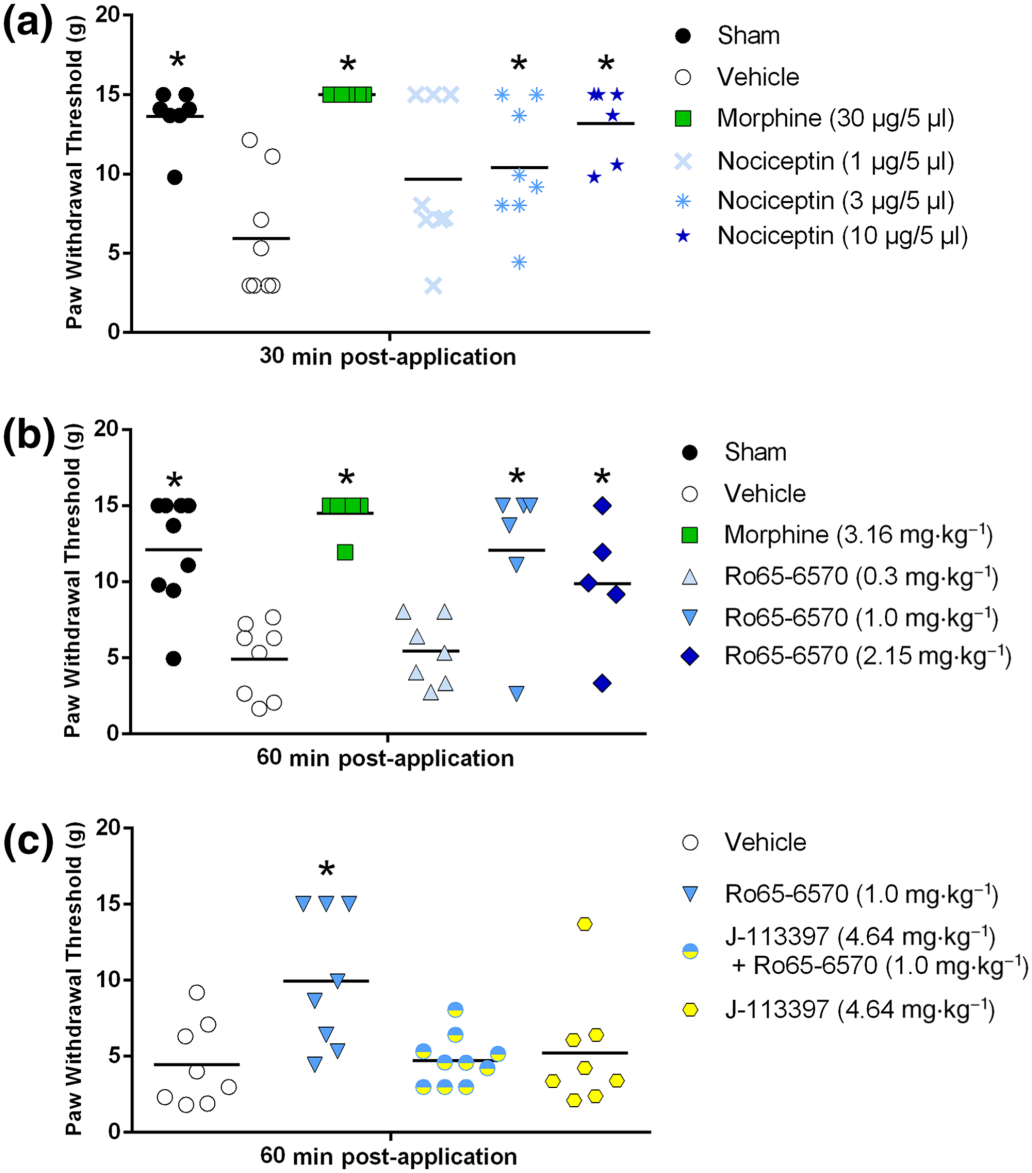
(a) The effect of nociceptin (i.t.) and morphine (i.t.) and (b) the effect of systemic Ro65‐6570 (i.p.; NOP receptor agonist) and morphine (i.p.) on mechanical allodynia in 1.5 × 10^6^·ml^−1^ of MRMT‐1/Luc2‐bearing animals in the von Frey test. (c) The effect of Ro65‐6570 (i.p.) is blocked by the NOP receptor antagonist J‐113397 (i.p.). All data are presented as mean ± SEM; *n* = 7 sham, *n* = 8 vehicle, *n* = 7 morphine, *n* = 8 nociceptin 1 μg, *n* = 8 nociceptin 3 μg, *n* = 6 nociceptin 10 μg in (a); *n* = 9 sham, *n* = 8 vehicle, *n* = 6 morphine, *n* = 7 Ro65‐6570 0.3 mg·kg^−1^, *n* = 6 Ro65‐6570 1.0 mg·kg^−1^, *n* = 5 Ro65‐6570 2.15 mg·kg^−1^ in (b); n = 9 vehicle, *n* = 8 Ro65‐6570, *n* = 8 J‐113397, *n* = 10 Ro65‐6570 + J‐113397 in (c); * *P* < .05 versus vehicle

The third experiment (Study 3, total *n* = 166) included three separate parts (A, B and C) to investigate the role of the NOP receptor system in cancer‐induced bone pain. To increase the behavioural test window, the cell quantity was increased by 0.25 × 10^6^·ml^−1^ to a final concentration of 1.5 × 10^6^·ml^−1^ of MRMT‐1/Luc2 cells. Based on the outcome of Study 2, all pharmacological experiments assessed mechanical allodynia by means of the von Frey hair test, 15 days post‐surgery. In Study 3A, the effect of i.t. nociceptin (1, 3, and 10 μg) was tested (30 min post‐administration), including i.t. morphine (30 μg) as positive control and vehicle (Figure 3a). In Study 3B, the effect of i.p. Ro65‐6570 (Rover, Wichmann, Jenck, Adam, & Cesura, 2000; 0.3, 1.0 and 2.15 mg·kg^−1^) was examined (60 min post‐administration), including i.p. morphine (3.16 mg·kg^−1^) as positive control and vehicle (Figure 3b). In study 3C, Ro65‐6570 (1.0 mg·kg^−1^, i.p.) was administered in combination with the selective NOP receptor antagonist J‐113397 (4.64 mg·kg^−1^, i.p.; Rutten, Schroder, Christoph, Koch, & Tzschentke, 2018; Figure 3c). The antagonist was administered 15 min prior to the agonist. Here, no morphine group was included, and sham animals were not subjected to behavioural testing. Blood samples were obtained to measure exposure levels of Ro65‐6570 and J‐113397 2 hr post‐administration. Additionally, bioluminescence signal images were obtained on Days 7, 13 and 19 post‐surgery, and X‐ray densitometry was performed at Day 19 post‐surgery (see Figure S2 and Data S2).

All behavioural tests and analyses were performed by an operator blinded to the experimental groups. Animals were randomly assigned to treatment groups by an operator unaware of the study hypothesis. Vehicle, positive control (morphine) and treatment doses were prepared in non‐transparent holders by an operator not involved in behavioural testing and administration of the treatment occurred in a randomized fashion. Finally, animals were killed using CO_2_ exposure at the end of the experiments, when the humane endpoint was reached, or killed by decapitation under isoflurane for blood sample collection.

Exclusion criteria for MRMT‐1/Luc2‐bearing animals were as follows: (a) if von Frey response at pretest was >9 g (*n* = 37) and (b) if no bioluminescence signal was present at day of behavioural testing (*n* = 23). Exclusion of animals occurred at the end of the experiments after behavioural and bioluminescence data analysis.

### 2.3 MRMT‐1 transfection with Luc2

The rat mammary gland carcinoma cell line, MRMT‐1 (Tohoku University, JP; TKG Cat# TKG 0132, RRID:CVCL_5156), was transfected with the luciferase (Luc2) gene (Trenzyme, Life Science Services, DE) to allow monitoring of cell presence and tumour growth over time. Parental MRMT‐1 cells were transfected by electroporation (Nucleofection) with target vector pExoIN7‐luc2P‐T2A. The target vector (pExoIN7‐luc2P‐T2A) contains the EF1α promoter sequence driving the equimolar expression of a puromycin resistance marker and the firefly luciferase (luc2P) gene. For transient transfection, 2 μg of target vector pExoIN7‐luc2P‐T2A‐copGFP was transfected into MRMT‐1 cells (Nucleofector II Device/program [A‐024], solution V). A selection process was started 24 hr post‐transfection with 3 μg·ml^−1^ of puromycin to select a stable transgenic cell pool. After cell expansion, the generated stable MRMT‐1_pExoIN7‐luc2P‐T2A cell pool was analysed for luciferase expression. Parental MRMT‐1 cells served as negative control.

### 2.4 Cell culture

The transfected MRMT‐1/Luc2 cells were cultured as previously described by Falk et al. (2015). Briefly, cells were cultured in RPMI 1640 medium (without phenol red) supplemented with 1% penicillin‐streptomycin and 10% heat‐inactivated FBS at least 2 weeks prior to use; cells were split every 2–3 days when cultivated and 2 days before surgery. On the day of surgery (Passage 21 ± 2; max. p23), cells were harvested with Detachin (Genlantis, San Diego, USA), re‐suspended in Hank Balanced Salt Solution (HBSS) to a final concentration of either: Study 1: 0.5 × 10^6^·ml^−1^, 1.0 × 10^6^·ml^−1^ and 1.25 × 10^6^·ml^−1^; Study 2: 1.25 × 10^6^·ml^−1^; and Study 3: 1.5 × 10^6^·ml^−1^, and kept on ice until use. All reagents were purchased from Thermo Fisher Scientific, DE.

### 2.5 Inoculation surgery

The inoculation of the MRMT‐1/Luc2 cells was performed as previously described by Falk, Schwab, et al. (2015). Briefly, approximately 6‐week‐old animals were anaesthetized using isoflurane (induction 4%; maintenance 2% ± 0.5% plus O_2_ 1.0 L·min^−1^; Baxter Deutschland GmbH, Unterschleissheim, DE) and placed on their dorsal side. The right hind limb was shaved and disinfected with 70% ethanol prior to exposing the anterior‐medial surface of the tibia by making a small (~1 cm) incision in the skin. A hole was made in the tibia using a 0.7‐mm drill bit (Fine Science Tools, Heidelberg, DE) to insert a catheter into the proximal intramedullary cavity. Ten microlitre of one of the four concentrations 0.5 × 10^6^·ml^−1^, 1.0 × 10^6^·ml^−1^, 1.25 × 10^6^·ml^−1^, or 1.5 × 10^6^·ml^−1^ of MRMT‐1/Luc2 cells in HBSS or vehicle (10‐μl HBSS) were injected using a 50‐μl Hamilton syringe, connected to the catheter. After removal of the catheter, the hole was closed using bone restorative material (IRM, Dentsply, PSG Procurement services GmbH, Lohmar, DE) and the wound was sutured (Vicryl sutur 4‐0, V292H, FS‐2S needle, 45 cm undyed, Johnson & Johnson medical GmbH, Ethicon Deutschland, Norderstedt, DE). Animals received carprofen, 5 mg·kg^−1^, s.c. (ReboPharm GmbH, Bocholt, DE) 24 hr pre‐surgery, peri‐operative and for two consecutive days post‐surgery and xylocaine (xylocaine pump spray, ReboPharm GmbH, Bocholt, DE) immediately after surgery and in the following 2 days post‐surgery.

### 2.6 Limb use test—Gait analysis

The limb use test was performed at baseline and on a daily basis starting on the first day after surgery. For the limb use test, animals were taken from their home cages, placed in an open space (60 cm × 120 cm) and allowed to move freely for 3 min for behavioural assessment (Falk, Al‐Dihaissy, Mezzanotte, & Heegaard, 2015). Scoring was as follows: 3: normal use of limb, 2: mild or insignificant limping, 1: significant limping and 0: no use of the affected limb. A limb use score of 0 was defined as a humane endpoint (25 animals were killed before the end of the study period [21 days post‐surgery] due to a broken leg, extra‐tibial tumour growth, injury after application, or when the limb use score reached 0 as a result of tumour progression).

### 2.7 Manual von Frey—Mechanical allodynia

Mechanical allodynia was measured using von Frey monofilaments (Ugo Basile, range 1–15 g), applied to the plantar surface of the ipsilateral hind paw. The paw withdrawal threshold was determined by means of the up‐and‐down principle, as described previously (Chaplan, Bach, Pogrel, Chung, & Yaksh, 1994). Briefly, animals were placed in a plastic box (L15 × W10.7 × H13.8 cm) located on a wire mesh platform, 30 min prior to first stimulation. Starting at 2 g, stimulation occurred in ascending intensity until a first paw withdrawal response. Consequently, stimulation was performed in descending intensity until no response was observed. Final positive versus final negative response defines the 50% g threshold followed by five consecutive stimulations followed in ascending or descending order, depending on a response. A cut‐off value of 15 g was used for cases where no withdrawal response was observed until that value. The 50% response withdrawal threshold was calculated using the formula: 50% g threshold = (10Xf + kδ)/(10.000), where Xf = value (log unit) of the final von Frey monofilament used; k = tabular value for the pattern of up and down responses; and δ = mean difference (in log units) between filaments. MRMT‐1/Luc2 animals with a value of >9 g and sham animals with a value <9 g were excluded from the analysis. The von Frey test was used to assess early alterations in cancer‐induced bone pain‐related behaviour as described previously by Falk, Schwab, et al. (2015) and excludes animals with an advanced cancer stage.

### 2.8 Weight bearing—Weight distribution

Weight distribution was determined using a rat incapacitance tester (Somedic Sales AB) as previously described by Schiene et al. with modifications as described by Falk et al. (Falk, Al‐Dihaissy, et al., 2015; Schiene, De Vry, & Tzschentke, 2011; Schott et al., 1994). Rats were placed in a Plexiglas chamber of the incapacitance tester with their front paws on an angled plate. Their hind paws were located on separate sensors to measure the weight distribution over 4 s in five trials. An average weight‐bearing ratio was calculated as follows: amount of weight of MRMT‐1/Luc2‐bearing limb/the total amount of weight placed on both limbs.

### 2.9 Cold plate—Cold allodynia

The test for cold allodynia was performed as previously described by Tzschentke, Linz, Frosch, and Christoph (2017) by using a metal plate (40 × 20 cm) that was cooled by a water bath to a constant temperature of 4°C. The metal plate was surrounded by a transparent Plexiglas box (height 30 cm) with a closed top. Animals were placed on the metal plate, and the number of paw withdrawal reactions was assessed over a time span of 2 min.

### 2.10 X‐ray densitometry—Bone density

Relative bone density was measured by X‐ray densitometry. Animals were anaesthetized with isoflurane (4% for induction; 2.5% for maintenance plus O2 1.0 L·min^−1^, Baxter Deutschland GmbH, Unterschleissheim, DE) and placed on the dorsal side in a Lumina XR apparatus (Caliper Life Sciences, Teralfene, BE), with the operated leg in the capture region. X‐ray images of the ipsilateral leg were captured and relative bone density was analysed using ImageJ (ImageJ 1.8.0_112; 64‐bit; ImageJ, RRID:SCR_003070). The mean greyscale value of a standard region of interest within the trabecular bone of the proximal tibia was measured, and the average of two corresponding background regions in the soft tissue proximal to tibia was subtracted. The relative greyscale value was then normalized to a standard aluminium wedge for each X‐ray image.

### 2.11 Luminescence imaging—Bioluminescence signal monitoring

d‐luciferin (Perkin Elmer, The Netherlands) dissolved in PBS (40 mg·kg^−1^, i.p.) was administered to the animals 10 min prior to measuring the bioluminescence signal. Animals were anaesthetized with isoflurane (4% for induction, 2.5% for maintenance plus O2 1.0 L·min^−1^, Baxter Deutschland GmbH, Germany) and placed on their dorsal side in a Lumina XR apparatus (Caliper Life Sciences, Teralfene, BE). Images of the ipsilateral limb were obtained using binning factor 4, exposure time 60 s and F/stop 1. The bioluminescence signal was analysed using the IVIS imaging software (Living Image©, version 4.0.0.9801; Caliper Life Sciences, Teralfene, BE) with a 25% threshold value of the signal. The total flux (photons per second) was used as a readout to calculate the mean bioluminescence signal. Also, the bioluminescence signal was used as a control and excluding criteria for animals: If MRMT‐1/Luc2‐inoculated animals did not show a bioluminescent signal from 16 days post‐surgery, animals were excluded from further analysis. In Figures S1 and S2, the y‐axis has been transformed in log‐formation as bioluminescent values are large and have a wide range; the data points themselves were not log‐transformed prior to drawing the figures.

### 2.12 Plasma sample collection

Blood samples were collected at the end of the experiment to perform biomarker analysis. Animals were deeply anaesthetized using isoflurane (induction 4%; maintenance 5% ± 0.5% plus O2 1.0 L min^−1^; Baxter Deutschland GmbH, Unterschleissheim, DE) and blood was obtained by heart punction via the left ventricle using a 20 Gauge S‐Monovette (needle connected to a l‐heparin filled S‐Monovette syringe [SARSTEDT AG & Co. KG, Nümbrecht, DE]); animals were killed after blood sampling by decapitation. To perform exposure analysis after compound administration, blood samples were obtained by punctuation of V. jugularis when animals were awake and manually fixed. The blood filled S‐Monovette syringe was then centrifuged at 12.000 RPM (25.758g) at 4°C for 10 min. Plasma samples were obtained and stored at −20°C for further analysis.

### 2.13 Biomarker analysis

Plasma samples obtained from six sham‐ and five vehicle‐treated MRMT‐1/Luc2‐bearing animals were analysed for inflammatory markers using a rat‐specific V‐Plex Pro‐inflammatory Panel 2 kit (analytes: IFN‐γ, IL‐10, IL‐13, IL‐1β, IL‐4, IL‐5, IL‐6, KC/GRO and TNF‐α; Meso Scale Discovery, Gaithersburg, MD, USA) according to manufacturer's instructions.

### 2.14 Tissue collection

Animals were killed at the end of the experiment via CO_2_ exposure and tissue, that is, ipsilateral DRGs (L4, L5, and L6) and ipsilateral bone marrow were harvested from sham and MRMT‐1/Luc2 animals. Tibias were dissected, and the bone was cut on the proximal end, close to the knee. Tibias were then centrifuged at 12.000 RPM for 3 s to let bone marrow flow out. Bone marrow and DRGs were collected in 0.5‐ml Eppendorf tubes. Tissue was snap‐frozen in liquid nitrogen immediately after harvesting and stored in −80°C for further use.

### 2.15 NOP receptor gene expression (RT‐PCR) analysis

Harvested rat DRGs and bone marrow were homogenized using a CryoMill (Retsch, Haan, DE) to keep samples frozen prior to thawing them in lysis buffer. RNA was isolated according to manufacturer's manual, including DNase digestion, using the RNeasy Mini Kit from Qiagen (Hilden, DE) and eluted with RNase‐free water for storage at −80°C for further use; concentrated RNA is eluted in 40‐μl RNase‐free water. Next, reversed transcription of RNA into cDNA was performed with 100‐ng RNA extracted from DRG and 200‐ng RNA extracted from bone marrow using the protocol from the high capacity cDNA reverse transcription kit (Applied Biosystems by Thermo Fisher Scientific, Darmstadt, DE). Briefly, 10 μl of RNA sample with 10‐μl RT master mix were mixed in wells of a 96‐well plate and loaded in the thermal cycler under the following conditions: 10 min at 25°C, 120 min at 37°C, 5 min at 85°C, and cool down to 4°C. Finally, RT‐PCR was conducted with 2‐μl cDNA (10 ng·μl^−1^) template in duplicates, mixed with TaqMan universal master mix II (with UNG; Thermo Fisher Scientific, Darmstadt, DE) and TaqMan Gene expression assay of the gene of interest: *oprl1* (NOP receptor, Rn00668206_g1; Applied Biosystems by Thermo Fisher Scientific, Darmstadt, DE) or the control gene of the TATA‐box binding protein (*Tbp*) gene which was selected for regenerating tissue, that is, bone marrow and used for standardization of samples (Rn01455646_m1, Applied Biosystems by Thermo Fisher Scientific, Darmstadt, DE). PCR conditions were as follows: First UNG incubation time (2 min at 50°C), then enzyme activation time (10 min at 95°C) and, finally, 40 cycles of denature (15 s at 95°C) and anneal/extend (1 min at 60°C); reaction volumes were 20 μl.

### 2.16 Drugs

The following drugs were used: morphine hydrochloride trihydrate (MacFarlan Smith Ltd, Edinburgh, UK), dissolved in 0.9% NaCl to a final dose of 300 μg per animal or 3.16 mg·kg^−1^, J‐113397 (Grünenthal GmbH, Aachen, DE), dissolved in 0.9% NaCl to a final dose of 4.64 mg·kg^−1^, Ro65‐6570 (Grünenthal GmbH, Aachen, DE), dissolved in 1% HPMC/0.5% Tween80 to final doses of 0.3, 1.0, or 2.15 mg·kg^−1^, and nociceptin (Tocris, Wiesbaden‐Nordenstadt, DE), dissolved in 0.9% NaCl to final doses of 1.0, 3.0, or 10.0 μg/animal. Intraperitoneal administration volume was 2 ml·kg^−1^, and i.t. administration volume was 5 μl per animal. The doses for Ro65‐6570 and J‐113397 are based on a previous publications showing selectivity of NOP receptor agonistic and antagonistic effect at these doses (Rutten et al., 2018). Nociceptin doses were based on previous publications using inflammatory and neuropathic pain models (Katsuyama et al., 2011; Ma et al., 2003), and morphine dose was based on historical in‐house data.

### 2.17 Statistics

Data analyses and plots were generated in GraphPad Prism 7.03 (Graph Pad Inc, CA, USA; GraphPad Prism, RRID:SCR_002798), except for the analysis of limb use data which was performed using SAS Studio 9.4.0.14150 (SAS Institute Inc., NC, USA; Statistical Analysis System, RRID:SCR_008567). All data are presented as mean ± SEM, and the level of significance was in all cases set to *P* < .05. The post hoc tests were conducted only if F in ANOVA achieved P < .05 and there was no significant variance inhomogeneity. The data and statistical analysis comply with the recommendations of the *British Journal of Pharmacology* on experimental design and analysis in pharmacology.

#### 2.17.1 Study 1

Mechanical hypersensitivity was analysed by one‐way ANOVA followed by Bonferroni method for correction of multiple comparisons. X‐ray images were analysed by Student's unpaired *t*‐tests and regression analysis was used to analyse bioluminescence signal images (data from Figure S1). Cytokine levels were analysed using Student's unpaired *t*‐test (data from Table 1). Gene expression analysis was performed using fold changes after normalization (data from Table 2).

#### 2.17.2 Study 2

Mechanical allodynia between ipsilateral and contralateral hind paws of sham and MRMT‐1/luc2‐bearing animals was analysed by Student's paired *t*‐test. The effect of morphine treatment in MRMT‐1/luc2‐bearing animals in weight bearing, cold plate, and von Frey was analysed using one‐way ANOVA followed by Dunnett's multiple comparisons test (data from Figure 2). Paw pressure test and open field data were analysed with two‐way ANOVA followed by Bonferroni multiple comparisons test.

#### 2.17.3 Study 3

The effect of compounds (morphine, nociceptin, Ro65‐6570 and J‐113397) were analysed by one‐way ANOVA followed by Dunnett's multiple comparisons for multiple comparisons (data from Figure 3). Corresponding limb use data were analysed with Friedmann's two‐way test followed by Wilcoxon's two sample test for post hoc analysis of individual time points. Regression analysis was used to analyse bioluminescence signals, and X‐ray images were analysed by Student's unpaired *t‐*tests (data from Figure S2).

### 2.18 Nomenclature statement of targets and ligands

Key protein targets and ligands in this article are hyperlinked to corresponding entries in http://www.guidetopharmacology.org, the common portal for data from the IUPHAR/BPS Guide to PHARMACOLOGY (Harding, Sharman et al., 2018), and are permanently archived in the Concise Guide to PHARMACOLOGY 2019/20 (Alexander et al., 2019).

## 3 RESULTS

### 3.1 Study 1—Cell quantity validation, inflammatory mediators, and gene expression

The different inoculated cell quantities each resulted in significantly reduced paw withdrawal thresholds; however, the most robust and consistent phenotype with stable bioluminescent signals, significant bone degradation, and minimal extinction of tumours (as assessed post‐mortem) was achieved using 1.25 × 10^6^·ml^−1^ and 1.5 × 10^6^·ml^−1^ of MRMT‐1‐/Luc2 cells (see Figure S1 and Data S1). The analysis of inflammatory cytokines in plasma, 21 days post‐surgery, showed no difference between sham‐ and vehicle‐treated MRMT‐1/Luc2‐bearing animals in the levels of IFN‐γ, IL‐13, IL‐1β, KC‐GRO and TNF‐α. A significant increase was found in the MRMT‐1/Luc2‐bearing animals for IL‐10, IL‐4, IL‐5 and IL‐6, compared to sham (see Table 1)

NOP receptor gene expression was examined in DRGs and bone marrow of MRMT‐1/Luc2‐bearing and sham animals (Table 2). CT values were normalized with the TATA‐binding box protein (*Tbp*), resulting in almost equal ΔCT values of NOP receptor expression in DRG, but different in bone marrow, resulting in a 69% lower NOP receptor expression in MRMT‐1/Luc2‐bearing animals compared to sham.

### 3.2 Study 2—Behavioural battery validation

Sham animals did not show a difference in von Frey withdrawal thresholds between ipsilateral and contralateral side, *t*(8) = 0.36. MRMT‐1/Luc2‐bearing animals had a significantly decreased ipsilateral paw withdrawal threshold compared to the contralateral side, *t*(12) = 8.29; data not shown.

Significant differences in weight‐bearing ratio were observed between preoperative baseline, pretest after operation and after morphine treatment. Dunnett's comparison showed that inoculation of MRMT‐1/Luc2 cells caused a significant reduction in weight‐bearing ratio compared to preoperative baseline animals and this was reversed 30, 60 and 90 min after morphine treatment (see Figure 2a). Significant differences in the number of reactions on the cold plate were observed between preoperative baseline, pretest after operation and after morphine treatment. Dunnett's comparison showed that inoculation of MRMT‐1/Luc2 cells caused a significant increase in the number of reactions on the cold plate compared to preoperative baseline animals and this was reversed 30 and 60 min after morphine treatment (see Figure 2b). Significant differences in von Frey withdrawal thresholds were observed between preoperative baseline, pretest after operation and after morphine treatment Dunnett's comparison showed that inoculation of MRMT‐1/Luc2 cells caused a significant decrease in von Frey withdrawal thresholds compared to preoperative baseline animals and this was reversed 30, 60 and 90 min after morphine treatment (see Figure 2c).

Paw withdrawal thresholds, as measured with the paw pressure test, did not differ between groups and MRMT‐1/Luc2‐bearing animals showed no difference in two parameters of the open‐field test (total distance and frequencies to enter the centre zone). Only the latency of MRMT‐1/Luc2‐bearing animals to enter the centre zone was significantly longer compared to sham animals (Table S1).

### 3.3 Study 3—Targeting the NOP receptor to study its involvement in cancer‐induced bone pain

The i.t. administration of nociceptin produced a significant dose‐dependent (ED_50_ = 1.76 μg; CI, [0.003, 4.250]) increase of paw withdrawal thresholds of 1.5 × 10^6^·ml^−1^ of MRMT‐1/Luc2‐bearing animals compared to vehicle‐treated MRMT‐1/Luc2‐bearing animals in mechanical allodynia (Figure 3a). Post hoc analysis showed a significant impairment of vehicle‐treated MRMT‐1/Luc2‐bearing animals compared to sham animals (Figure 3a) and morphine fully reversed this impairment (Figure 3a). In addition, nociceptin 1 μg did not alleviate mechanical allodynia (Figure 3a), while 3 μg and 10 μg did (Figure 3a). Also, morphine significantly reversed mechanical allodynia compared to 1‐ and 3‐μg nociceptin but showed no difference with 10‐μg nociceptin (Figure 3a).

In the experiment with systemic administration (i.p.) of the NOP receptor agonist Ro65‐6570, vehicle‐treated MRMT‐1/Luc2‐bearing animals showed a significantly reduced paw withdrawal threshold compared to sham‐treated animals (Figure 3b:). Treatment of the MRMT‐1/Luc2‐bearing animals with Ro65‐6570 or morphine produced a significant increase in paw withdrawal threshold (Figure 3b) and post hoc analysis showed no effect with 0.3 mg·kg^−1^ of Ro65‐6570 (Figure 3b), while 1.0 and 2.15 mg·kg^−1^ attenuated mechanical allodynia (Figure 3b:). However, the dose of 2.15 mg·kg^−1^ produced sedative side effects, which may confound the behavioural readout. The positive control morphine fully reversed mechanical allodynia in MRMT‐1/Luc2‐bearing animals (Figure 3b). Also, morphine significantly reversed mechanical allodynia compared to 0.3 mg·kg^−1^ but showed no difference with 1.0 and 2.15 mg·kg^−1^ of Ro65‐6570 (Figure 3b).

In the antagonist study, we found a treatment effect between the groups (Figure 3c) and post hoc analysis showed that Ro65‐6570 significantly attenuated mechanical allodynia in MRMT‐1/Luc2‐bearing animals compared to sham controls (Figure 3c). This effect was blocked when Ro65‐6570 was administered together with J‐113397 (Figure 3c). The antagonist alone had no effect on mechanical allodynia compared to sham controls (Figure 3c). Exposure analysis of terminal plasma samples showed a concentration of 0.05 μmol·L^−1^ for Ro65‐6570 1.8 hr post‐administration and a concentration of 0.177 μmol·L^−1^ for J‐113397 2 hr post‐administration.

The difference in limb use score, bioluminescent signals and relative bone density were only assessed between the sham and MRMT‐1/Luc2‐bearing animals from Study 3A–C to monitor tumour presence, analysis of bone degradation and as human endpoint respectively. The data provide an illustration for the consistency and robustness of our model and is shown in Figure S2A–C, S2D–F, and S2G–I.

## 4 DISCUSSION

Every year, close to 10 million people are diagnosed with cancer and this is accompanied by pain in 30% to 50% of patients with advanced cancers (Wiffen, Wee, Derry, Bell, & Moore, 2017). However, it is suggested that half of these patients receive inadequate therapy for pain control (Schug & Chandrasena, 2015). Several animal models of cancer‐induced bone pain have been developed to further understand its mechanisms. An example is the intratibial inoculation of MRMT‐1 cells in Sprague Dawley rats (Medhurst et al., 2002). Accordingly, our study shows that inoculation of 1.25 × 10^6^·ml^−1^ and 1.5 × 10^6^·ml^−1^ of MRMT‐1/Luc2 cells results in a reliable and robust cancer‐induced bone pain‐related phenotype with a progressive development of the disease. Similar to the literature, the bioluminescent signal plateaus after the initial 10 days and it was suggested that lack of oxygen renders the core of the tumour necrotic and limits the chemical reaction of luciferase to produce the bioluminescent signal (Appel et al., 2017; Diaz‐delCastillo et al., 2018). Although the required cell quantity in our lab was higher in comparison to previous studies (Falk, Al‐Dihaissy, et al., 2015), the range of inoculated cell number and the behavioural outcome in pain phenotype is robust and comparable with other literature (Medhurst et al., 2002; Schwei et al., 1999). The up‐regulation of IL‐4, IL‐5 and IL‐10 as seen in our MRMT‐1/Luc2‐bearing animals is in agreement with previous data and is indicative for cancer physiology modulation (Goldstein et al., 2011; Rosen et al., 1998). In addition, IL‐6 is suggested to play a substantial role in the development of pathological pain conditions, for example, cancer pain (Zhou et al., 2016) by sensitizing nociceptive fibres and mediating peripheral and central sensitization of DRG neurons via transient receptor potential cation channel subfamily V member 1 (TRPV1) receptors (Fang et al., 2015; Remeniuk et al., 2018). Next, little is known about the presence of the NOP receptor in the musculoskeletal tissue. Data from synovial tissue are contradictory (Kumar et al., 1999; Zhang & McDougall, 2006) but suggest that NOP receptor is present in tendon and joint tissue (Ackermann et al., 2001; McDougall, 2003). This study is the first to describe NOP receptor gene expression in rat bone marrow. Interestingly, NOP receptor expression was significantly decreased in bone marrow of MRMT‐1/Luc2‐bearing animals, possibly due to invading tumour cells diminishing NOP receptor expressing cells. Further studies investigating the presence of the NOP receptor in bone, cartilage or synovium are warranted, as this yields valuable information for peripheral pain management, for example, intra‐articular treatment for joint‐associated pain and bone cancer (Bergstrom et al., 2006).

Our behavioural battery identified ipsilateral mechanical allodynia (measured by the von Frey test) as leading phenotype which could be fully reversed by morphine, while cold plate and weight‐bearing readouts showed a more moderate phenotype. Cold allodynia has been described in different rodent models of chronic pain (Linz et al., 2014) but appears to be much less pronounced than mechanical allodynia in our cancer‐induced bone pain model. Weight bearing has been previously described as a possible readout in cancer‐induced bone pain models (Medhurst et al., 2002). The test window for both cold allodynia and weight bearing in our MRMT‐1/Luc2‐bearing animals was too small to allow detailed pharmacological investigations, although the cancer‐induced change in both readouts was partially reversed by morphine. Furthermore, we did not observe a cancer‐induced bone pain phenotype using the paw pressure test or when using the open field test as an alternative readout for non‐evoked pain‐like behaviour. Taken together, the full battery of behavioural testing revealed the von Frey test as the most robust readout in this model of cancer‐induced bone pain.

In order to investigate the role of the NOP receptor system in cancer‐induced bone pain, selective agonists and an antagonist were tested in the MRMT‐1/Luc2 model using mechanical allodynia as a readout. We observed full reversal of mechanical allodynia in the von Frey test by both morphine and NOP agonists. Intrathecal nociceptin produced a dose‐dependent anti‐allodynic effect. Previously, i.t. nociceptin injections were shown to have anti‐nociceptive effects in tail‐flick (acute pain; Tian et al., 1997; Wang, Zhu, Cao, & Wu, 1999) and anti‐hypersensitive actions in rat models of neuropathic and inflammatory pain (Courteix et al., 2004; Fu et al., 2006; Hao, Xu, Wiesenfeld‐Hallin, & Xu, 1998; Ju, Shin, Na, & Yoon, 2013; Katsuyama et al., 2011; Ma et al., 2003). Spinal NOP receptor activation has been shown to be efficacious in both neuropathic and inflammatory pain models (Schroder et al., 2014). Overall, NOP receptor activation is suggested to have more potent effects in chronic pain as compared to acute pain conditions in rodents (Schroder et al., 2014). Accordingly, the potency of nociceptin in our cancer‐induced bone pain model is in the same range as the potency observed in other chronic pain models (Courteix et al., 2004; Fu et al., 2006; Hao et al., 1998; Ju et al., 2013; Lee, 2013; Ma et al., 2003).

The NOP receptor agonist Ro65‐6570 significantly reversed mechanical allodynia at a dose of 1.0 mg·kg^−1^ but showed sedative side effects at a higher dose. This is consistent with what has been described in a rat mononeuropathic spinal nerve ligation model (Rutten et al., 2018). The analgesic effect of 1.0 mg·kg^−1^ of Ro65‐6570 was blocked by 4.64 mg·kg^−1^ of J‐113397, a NOP receptor antagonist. As we have previously shown that these doses of both compounds are selective for the NOP receptor (Rutten et al., 2018), this finding implies that the analgesic effect of Ro65‐6570 was NOP receptor mediated. Several studies support analgesic efficacy of NOP receptor activation in preclinical models of pain, for example, chronic constriction injury (Wu & Liu, 2018), spinal nerve ligation (Rutten et al., 2018) and complete Freund's adjuvant (Chen & Sommer, 2007). Moreover, a possible involvement of the NOP receptor in cancer‐induced bone pain has been suggested by the efficacy of cebranopadol, a mixed opioid/NOP receptor agonist (Linz et al., 2014), and buprenorphine, a mixed μ‐opioid peptide/NOP partial agonist and κ‐opioid peptide/δ‐opioid peptide antagonist (Gastmeier & Freye, 2009) in rats. However, the present study is the first to reveal highly efficacious and potent relief of cancer‐induced bone pain by selectively targeting the NOP receptor in rats.

It has previously been described that spinal and peripheral activation of the NOP receptor contributes to analgesic effects, whereas supraspinal activation may result in hyperalgesia (Schroder et al., 2014). The exact mechanism behind this dichotomy remains unclear, but it is speculated that i.t. NOP receptor activation attenuates mechanical allodynia via hyperpolarization of (glycinergic) interneurons which are disinhibited upon injury, thereby enabling touch sensation to engage with nociceptive projection neurons (Ozawa et al., 2018).

On the other hand, the hyperalgesic effects of supraspinal NOP receptors are suggested to be a result of the inhibition of OFF cells within the rostral ventromedial medulla (Schroder et al., 2014). Descending pathways from the rostral ventromedial medulla to the spinal cord dorsal horn inhibit ascending nociceptive signals (Lau & Vaughan, 2014), and supraspinal NOP receptor activation may thus result in disinhibition at the spinal level (Schroder et al., 2014). Determination of the binding affinity of nociceptin at the NOP receptor has been notoriously difficult and reported K_i_ values vary widely (0.03–2 nM) due to differences in binding assays and unusual binding properties of nociceptin (see Dooley & Houghten, 2000). Furthermore, differences may exist in the binding affinity of nociceptin in the spinal cord and brain (Kusaka, Yamada & Kimura, 2001). Here, full efficacy was observed after spinal application of 10‐μg (~5.5 nM) nociceptin, which should cover the NOP receptor spinally 2.5‐ to 18‐fold. The free plasma concentrations of Ro65‐6570 (50 nM; Ki_NOP_ = 0.84 nM) and J‐113397 (180 nM; Ki_NOP_ = 0.80 nM) represent a respective 50‐ and 20‐fold coverage of NOP receptors (Ki_NOP_ values are unpublished in house scintillation proximity assay results [personal communication, Thomas Koch]). After systemic administration, both peripheral and central NOP receptors are likely to be affected, and since fraction unbound in brain and KP_u,u_ for Ro65‐6570 and J‐113397 are not known, the observed activity may be due to activation of central and/or peripheral NOP receptors.

In conclusion, this study is the first to demonstrate a potential benefit of targeting the NOP receptor either spinally or systemically to alleviate cancer‐induced bone pain, and to show the expression of NOP receptor in bone marrow in rodents. Targeting the NOP receptor may result in potent analgesia and selective NOP receptor agonists may have a beneficial side effect profile (see Schroder et al., 2014) compared to classical μ‐opioids in cancer‐induced pain. Further research into selective and potent NOP agonists is highly warranted.

## CONFLICT OF INTEREST

S.H.J.S, J.K., T.C., T.M.T., and K.R. are employees of Grünenthal GmbH. M.D.‐dC. and A.‐M.H. have no conflict of interest.

## AUTHOR CONTRIBUTIONS

According to the guidelines for Authorship policy as stated within the “Author Guidelines for *British Journal of Pharmacology*,” the authors S.H.J.S, J.K., T.C., M.D.‐dC., A.‐M.H., and K.R. made substantial contributions regarding conception and design, acquisition of data, analysis, and interpretation of data; the authors S.H.J.S., T.C., T.M.T., M.D.‐dC., A‐M.H., and K.R. were involved in drafting and revising the manuscript for critically important intellectual content; all authors as listed in the manuscript, S.H.J.S., J.K., T.C., T.M.T., M.D.‐dC., A.‐M.H., and K.R., provided final approval for publishing and agreed to be accountable for all aspects of the work.

## DECLARATION OF TRANSPARENCY AND SCIENTIFIC RIGOUR

This Declaration acknowledges that this paper adheres to the principles for transparent reporting and scientific rigour of preclinical research as stated in the BJP guidelines for Design & Analysis, and Animal Experimentation, and as recommended by funding agencies, publishers and other organisations engaged with supporting research.

## Supporting information

Figure S1. Supporting Information

Figure S2. Supporting Information

Table S1. The results of the behavioural tests from Study 2B, paw pressure test and open field, measured 18‐20 days post‐surgery in sham and 1.5 x 10^6^/ml MRMT‐1/Luc2‐bearing animals. Data is represented as mean ± SEM; n for sham = 8; n for MRMT‐1/Luc2‐bearing animals = 13; * p<0.05 vs sham

Data S1. Supporting Information
