# Supplementary figures and images for: The nociceptin/orphanin FQ receptor system as a target to alleviate cancer‐induced bone pain in rats: Model validation and pharmacological evaluation
Source: Br J Pharmacol. 2020 Jan 21;178(9):1995–2007. doi: 10.1111/bph.14899 (PMC8246843; doi:10.1111/bph.14899)

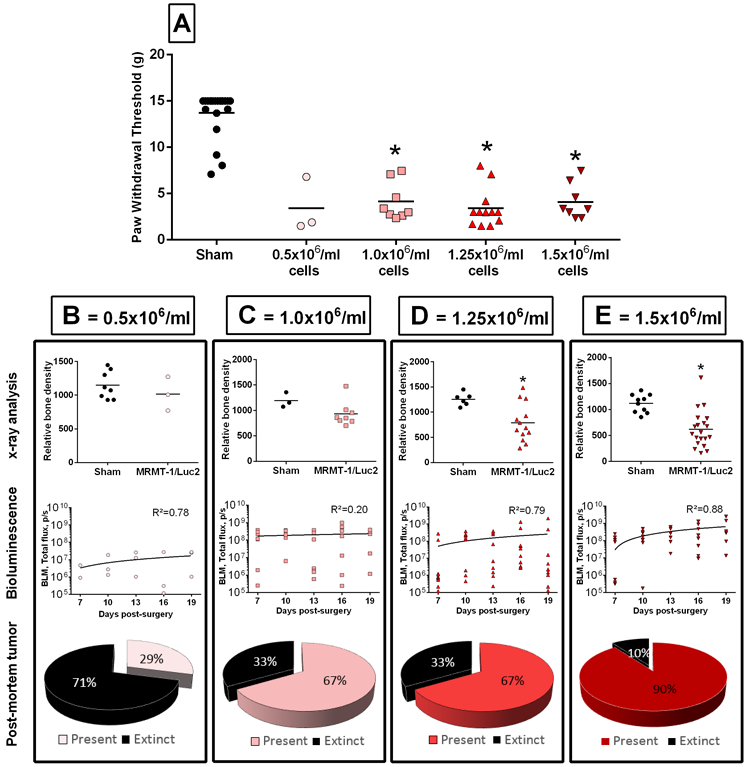

Supplement: Supplementary file 1 — Figure S1. Supporting Information [file BPH-178-1995-s004.png]

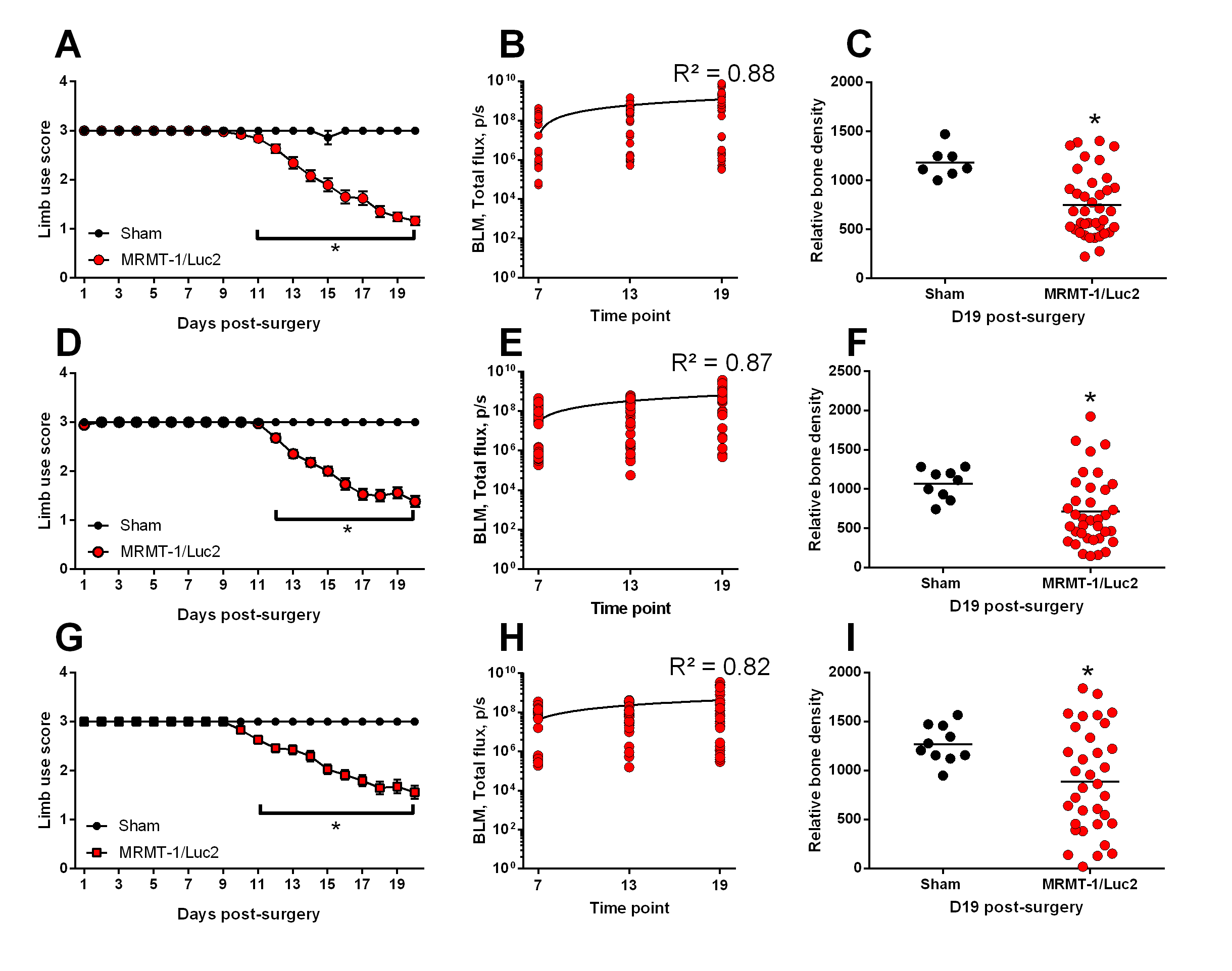

Supplement: Supplementary file 2 — Figure S2. Supporting Information [file BPH-178-1995-s002.png]
