# Supplementary material for: The nociceptin/orphanin FQ receptor system as a target to alleviate cancer‐induced bone pain in rats: Model validation and pharmacological evaluation
Source: Br J Pharmacol. 2020 Jan 21;178(9):1995–2007. doi: 10.1111/bph.14899 (PMC8246843; doi:10.1111/bph.14899)
Supplement: Supplementary file 3 — Table S1. The results of the behavioural tests from Study 2B, paw pressure test and open field, measured 18‐20 days post‐surgery in sham and 1.5 x 10^6^/ml MRMT‐1/Luc2‐bearing animals. Data is represented as mean ± SEM; n for sham = 8; n for MRMT‐1/Luc2‐bearing animals = 13; * p<0.05 vs sham [file BPH-178-1995-s001.docx]

**Supplementary table 1. The results of the behavioural tests from Study 2B, paw pressure test and open field, measured 18-20 days post-surgery in sham and 1.5 x 10^6^/ml MRMT-1/Luc2-bearing animals. Data is represented as mean ± SEM; n for sham = 8; n for MRMT-1/Luc2-bearing animals = 13; * p<0.05 vs sham.**

| **Behavioural tests Study 2B** | | |  |
| --- | --- | --- | --- |
| **Test** | **Sham mean ± SEM** | **MRMT-1/Luc2 mean ± SEM** | **Statistics** |
| Paw pressure test (g) | 162.0 ± 12.84 | 177.92 ± 11.03 | F (1, 19) = 0.95  p = 0.34 |
| Open field  – Total distance (m) | 3.46 ± 0.63 | 2.48 ± 0.63 | F (1, 18) = 2.03 p = 0.17 |
| Open field  – Centre zone entry (frequency) | 11.86 ± 2.31 | 8.25 ± 2.26 | F (1, 17) = 1.06 p = 0.32 |
| Open field  – Centre zone entry (latency in s) | 29.34 ± 8.73 | 159.93 ± 50.07 | F (1, 17) = 3.40 * p = 0.08 |
