# Supplementary material for: The nociceptin/orphanin FQ receptor system as a target to alleviate cancer‐induced bone pain in rats: Model validation and pharmacological evaluation
Source: Br J Pharmacol. 2020 Jan 21;178(9):1995–2007. doi: 10.1111/bph.14899 (PMC8246843; doi:10.1111/bph.14899)
Supplement: Supplementary file 4 — Data S1. Supporting Information [file BPH-178-1995-s003.docx]

**Supporting information**

Supplementary data 1

Study 1 showed a significant reduction in paw withdrawal thresholds (von Frey) with each inoculated cell quantity (Supplementary Figure 1A: (F (4, 47) = 59.92), p < 0.0001; vs sham operated animals: t(47)_1.0x10_^6^_/ml MRMT-1/Luc2_ = 10.13, p < 0.0001; t(47)_1.25x10_^6^_/ml MRMT-1/Luc2_ = 12.51, p < 0.0001; t(47)_1.5x10_^6^_/ml MRMT-1/Luc2_ = 10.18, p < 0.0001). However, relative bone density decreased substantially when 1.25x10^6^/ml (Supplementary Figure 1D: (F (12, 5) = 8.79), p = 0.026; t(17) = 2.95, p = 0.009) and 1.5x10^6^/ml MRMT-1/Luc2 (Supplementary Figure 1E: (F (19, 9) = 4.07), p = 0.036; t(17) = 4.22, p = 0.002) cells were inoculated, but not at the lower quantities of 0.5x10^6^/ml (Supplementary Figure 1B: (F (2, 7) = 1.48), p = 0.58; t(9) = 0.88, p = 0.40) and 1.0x10^6^/ml (Supplementary Figure 1C: (F (7, 2) = 2.73), p = 0.59; t(9) = 1.75, p = 0.11) MRMT-1/Luc2 cells.

Accordingly, the bioluminescent signal showed an increased correlated signal over the duration of the experiment by the inoculation of 1.0x10^6^/ml MRMT-1/Luc2 cells (Supplementary Figure 1C: (F (1, 3) = 0.76), p = 0.45) compared to 0.5x10^6^/ml MRMT-1/Luc2 cells (Supplementary Figure 1C: (F (1, 3) = 10.61), p = 0.05), but reaches a plateau at quantities >1.0x10^6^/ml (for 1.25x10^6^/ml Supplementary Figure 1D: (F (1, 3) = 11.57), p = 0.04, R^2^ = 0.79; for 1.5x10^6^/ml Supplementary Figure 1E: (F (1, 3) = 15.11), p = 0.03, R^2^ = 0.83). The proportion of animals that developed an extraosseous tumour (tested by post-mortem dissection of the ipsilateral limb was lowest at 0.5x10^6^/ml MRMT-1/Luc2 cells, equal between 1.0x10^6^/ml and 1.25x10^6^/ml MRMT-1/Luc2 cells, and largest after inoculation of 1.5x10^6^/ml MRMT-1/Luc2 cells (used in Study 3).

<INSERT SUPPLEMENTARY FIGURE 1>

Supplementary data 2

In all three experiments the inoculation of MRMT-1/Luc2 cells resulted in a time-dependent decrease in limb use scores (Supplementary Figure 2A: (F (44) = 5.99), p < 0.0001; Supplementary Figure 2D: (F (41) = 6.30), p < 0.0001; Supplementary Figure 2G (F (45) = 8.07), p < 0.0001), showed increased bioluminescence signals over time (Supplementary Figure 2B: (F (1, 1) = 6.3), p = 0.24, R² = 0.86; Supplementary Figure 2E: (F (1, 1) = 6.7), p = 0.24, R² = 0.87; Supplementary Figure 2H: (F (1, 1) = 4.4), p = 0.28, R² = 0.81) while bone density decreased (Supplementary Figure 2C: t(42) = 2.9, p = 0.006; Supplementary Figure 2F: t(39) = 2.7, p = 0.01; Supplementary Figure 2I: t(43) = 3.0, p = 0.005).

<INSERT SUPPLEMENTARY FIGURE 2>

<INSERT SUPPLEMENTARY TABLE 1>

**Paw pressure test – mechanical hyperalgesia**

The paw pressure test measuring hyperalgesia was conducted using an analgesia-meter (**Ugo** Basile, Italy) as previously described by Randall and Selitto (1957). Gradually increasing pressure was applied on the dorsal side of the hind paw from the operated limb via a cone-shaped ending with a round tip. The force (in grams) necessary to elicit paw withdrawal (representing a nociceptive response) reflects the nociceptive paw withdrawal threshold. A cut-off value of 250 g was used as maximum pressure to prevent damage at the paw. Animals were habituated to the paw pressure apparatus by walking freely on the apparatus 1 minute prior to testing. During testing the animal was gently fixed by the hand of the operator.

**Open field test – locomotor and explorative behaviour**

The open field apparatus consisted of a black wooden arena (100 x 100 x 40 cm), located in the corner of the experimental room which was luminated with red light. A camera was placed above the arena, capturing only the arena area. Via Ethovision tracking software (Noldus, Groningen, the Netherlands) the arena was virtually divided in a centre area (30 x 30 cm, in the middle of the arena) and the walls area (space outside the centre area). Rats were individually placed in a corner of the arena and were allowed to move freely for 10 min. The midpoint of the body was automatically tracked by the Ethovison software (Noldus, Groningen, the Netherlands). The parameters analysed were total distance moved, times entering the centre zone and latency before entering the centre zone.
